# Incidence, Characteristics, and Management of Patients with Recurrent Myocardial Infarctions: Insights from the EYESHOT POST-MI

**DOI:** 10.1155/2022/4593325

**Published:** 2022-03-18

**Authors:** Leonardo De Luca, Furio Colivicchi, Domenico Gabrielli, Donata Lucci, Gabriele Grippo, Francesco Piemonte, Bruno Villari, Andrea Di Lenarda, Fabrizio Oliva, Michele Massimo Gulizia

**Affiliations:** ^1^Department of Cardiosciences, Division of Cardiology, A.O. San Camillo-Forlanini, Roma, Italy; ^2^Division of Cardiology, S. Filippo Neri Hospital, Roma, Italy; ^3^ANMCO Research Center, Heart Care Foundation, Firenze, Italy; ^4^Division of Cardiology, Nuovo Ospedale, Prato, Italy; ^5^Division of Cardiology, San Giovanni di Dio Hospital, Frattamaggiore (NA), Italy; ^6^Division of Cardiology, Ospedale Fatebenefratelli, Benevento, Italy; ^7^Division of Cardiology, Azienda Sanitaria Universitaria Integrata di Trieste, Trieste, Italy; ^8^Cardiac Intensive Care Unit and De Gasperis Cardio Center, ASST Grande Ospedale Metropolitano Niguarda, Milano, Italy; ^9^Division of Cardiology, Garibaldi-Nesima Hospital, Catania, Italy

## Abstract

**Background:**

It is unknown whether patients who survived two or multiple episodes of myocardial infarction (MI) present different clinical characteristics and management than patients at their first MI.

**Methods:**

The EYESHOT post-MI was a prospective, observational, nationwide study aimed to evaluate the management of patients presenting to cardiologists 1 to 3 years from the last MI event. In 3 months of enrolment, 165 Italian cardiology centers included 1633 consecutive post-MI patients. In the present analysis, we stratified the study cohort according to the number of prior MI episodes (i.e., 1, 2 or ≥3).

**Results:**

Among the 1618 patients enrolled with complete data on MI history, 1335 (82.5%) were at their first MI episode, 209 (12.9%) had a history of 2 MIs, and the remaining 74 (4.6%) had ≥ 3 prior MIs. Patients with a history of multiple MIs were increasingly older and presented a significantly higher rate of risk factors compared to those at their first MI. During the year prior to enrolment, patients with 2 or ≥3 MI episodes more frequently underwent coronary angiography compared to the other group (*p* < 0.0001). In addition, several lifesaving and antianginal drugs were more frequently prescribed in patients presenting with a history of multiple MIs compared to those at their first MI.

**Conclusions:**

Our data suggest that patients with multiple MIs managed by cardiologists in routine clinical practice present an incremental clinical risk, more frequently undergo coronary angiography, and are more intensively managed with pharmacological therapies compared to patients at their first MI episode.

## 1. Introduction

Survivors of acute myocardial infarction (MI) have a considerable risk of recurrent infarction after discharge, especially during the first year [1–8]. Patients who experience re-MI are exposed to an increased risk of all-cause and cardiovascular major events, including mortality [1–8]. For this reason, the identification of re-MI predictors, as well as the intensification of secondary prevention strategies in patients at higher risk, appears crucial to reduce long-term cardiovascular events [9]. To date, it is unknown whether patients who survived two or multiple episodes of MI present clinical characteristics of greater risk and receive more aggressive pharmaceutical treatments than patients at their first MI.

Using the data of the EYESHOT (EmploYEd antithrombotic therapies in patients with acute coronary Syndromes HOspitalized in Italy) Post-MI study [10] that included consecutive post-MI patients presenting to cardiologists, we sought to assess incidence, characteristics, and current management of patients at their first MI compared to those with a history of multiple MIs.

## 2. Methods

The EYESHOT Post-MI was a prospective, observational, nationwide registry of consecutive patients with a prior MI managed by cardiologists [10]. All patients admitted in cardiology units and/or ambulatory clinics during a period of 3 months with a documented history of presumed spontaneous MI event (non-ST elevation, NSTEMI, or ST-elevation-MI, STEMI) occurred between 1 and 3 years before enrolment have been included. We excluded patients aged <18 years and those not giving informed consent. Enrolment was made at the beginning of outpatient or day-hospital visit or at hospital admission [10].

No specific protocols for evaluation, management, and/or treatment have been put forth during this observational study. However, current guidelines for the management of STEMI and NSTE-ACS [11, 12] have been discussed during the investigator meetings.

All patients were informed of the nature and aims of the study and asked to sign an informed consent for the anonymous management of their individual data. Local Institutional Review Boards (IRB) approved the study protocol according to the current Italian rules.

Each site started patient enrolment after local IRB approval. Therefore, data were collected in different periods of consecutive 3 months in each site between March 1^st^, 2017, and December 16^th^, 2017 [10, 13]. Over these periods, 1633 consecutive patients were enrolled in 165 cardiology centers. In the present analysis, we considered 1618 patients (99.1%) with complete data on prior MI history.

### 2.1. Data Collection and Data Quality

Data on baseline characteristics, including demographics, risk factors, and medical history, were collected. Information on the use of diagnostic cardiac procedures, type and timing of revascularization therapy (if performed), and use of pharmacological or nonpharmacological therapies were recorded on an electronic case report form (CRF) [10].

At each site, the principal investigator was responsible for screening consecutive patients presenting between 1 and 3 years from the last MI. Data were collected using a web-based, electronic CRF with the central database located at the ANMCO (Associazione Nazionale Medici Cardiologi Ospedalieri) Research Center. By using a validation plan, integrated in the data entry software, data were checked for missing or contradictory entries and values out of the normal range.

### 2.2. Statistical Analysis

Categorical variables are presented as number and percentages and compared by the Chi-square test. Continuous variables are presented as mean and standard deviation (SD), except for timing from last MI and triglycerides, which are reported as median and interquartile range (IQR), and were compared by the analysis of variance (ANOVA), if normally distributed, or by Kruskall–Wallis test, if not. The study cohort was stratified according to the number of prior MI episodes (i.e., 1, 2, or ≥3).

A *p* value < 0.05 was considered statistically significant. All tests were 2-sided. Analyses were performed with SAS system software, version 9.4.

## 3. Results

Among the 1618 patients enrolled with complete data on MI history, 1335 (82.5%) were at their first MI episode, 209 (12.9%) had a history of 2 MIs, and the remaining 74 (4.6%) had ≥3 prior MIs.

Baseline characteristics of the study population are shown in Table 1. The mean age of enrolled patients was 66 ± 12 years, 80% were male, 28% diabetics, and 74% had hypercholesterolemia. The mean ejection fraction was 52 ± 10%, a systolic blood pressure ≤140 mmHg was present in 82%, a heart rate ≤70 bpm in 71%, and low-density lipoprotein (LDL) cholesterol levels ≤70 mg/dl in 49% of cases. Patients with a history of 2 or ≥3 MIs were older and presented a significantly higher rate of risk factors such as diabetes mellitus, hypertension, hypercholesterolemia, history of atrial fibrillation, heart failure, cerebrovascular events, surgical myocardial revascularization, chronic kidney disease or peripheral artery disease and higher levels of creatinine, and a lower mean left ventricular ejection fraction compared to those at their first MI (Table 1).

### 3.1. Diagnostic Procedures and Pharmacological Treatments

**Figure 1:**
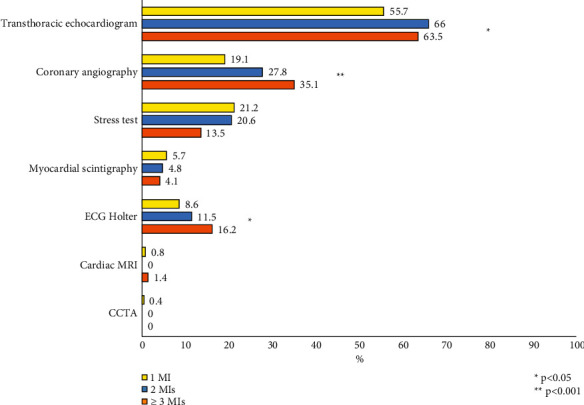
Diagnostic procedures performed within 12 months before enrolment in the 3 groups. CCTA : coronary computed tomography angiography; MRI : magnetic resonance imaging.

**Figure 2:**
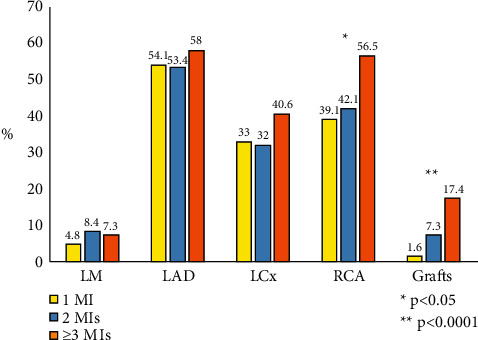
Frequency of coronary vessel with significant stenoses (≥50% for left main and ≥70% for other vessels at coronary angiography) in the 3 groups (data on 1456 patients with angiographic data available). LAD: left anterior descending; LCx: left circumflex; LM: left main; RCA: right coronary artery.

Diagnostic cardiac procedures performed in the 3 groups within the previous 12 months from enrolment are shown in Figure 1. During the year prior to enrolment, most patients received a transthoracic echocardiogram, followed by coronary angiography. Patients with ≥3 MI episodes more frequently underwent a coronary angiography and a Holter ECG compared to the other groups (Figure 1). Coronary angiography was performed in 1581 patients (97.7%), and, among these, 1456 (92.1%) had the angiographic data available (92.8% for those with 1 prior MI episode, 87.3% for those with 2 MI episodes, and 93.2% for patients with ≥3 MIs). Patients with a history of ≥3 MIs presented a higher rate of multivessel coronary artery disease (47.8% vs 38.2% vs 31.8%; *p* = 0.008), a higher number of coronary stent implanted (Table 1), and significant stenoses of the right coronary artery and of venous or arterial grafts compared to other groups (Figure 2).

Among patients enrolled, 15.3% were on diet, a regular physical activity was performed by 28.3%, without a significant difference between the 3 groups, and 61.0% of smokers at the time of last MI declared to have stopped smoking, with a higher prevalence in those at their first MI episode compared to other groups (*p* < 0.0001).

**Figure 3:**
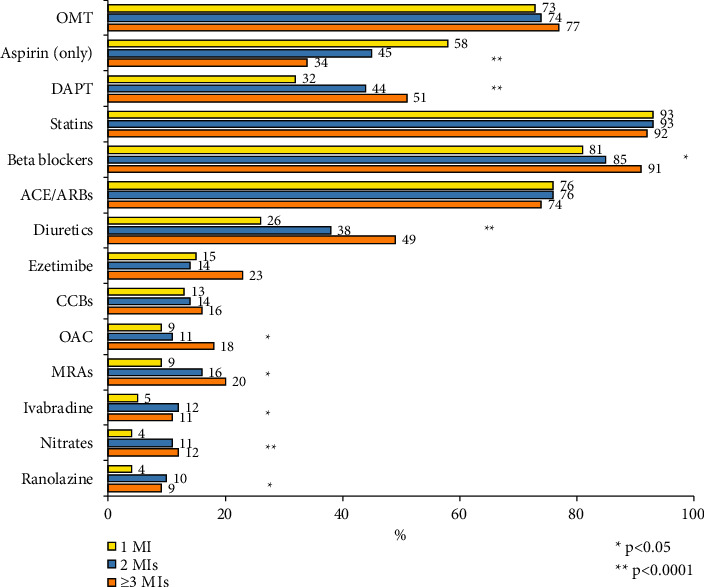
Cardiovascular therapies prescribed at enrolment in the 3 groups.

At the time of enrolment, most patients were prescribed on statins (93%), followed by beta-blockers (82%) and angiotensin-converting enzyme inhibitors/angiotensin receptor blockers (76%). Beta-blockers, diuretics, dual antiplatelet therapy (DAPT), mineralocorticoid receptor antagonists, oral anticoagulants, and antianginal drugs were more frequently used among patients presenting with a history of 2 or ≥3 MIs compared to those presenting at their first MI (Figure 3).

## 4. Discussion

The present study provides unique contemporary data on clinical characteristics, healthcare resource utilization, and treatment patterns of patients at their first MI or with multiple MIs managed by cardiologists in routine clinical practice. Although several studies have evaluated the impact of re-MI on outcomes [1–9], this is the first study, to the best of our knowledge, that has demonstrated the incremental association between clinical risk and the increase in the number of re-MI events. Patients with multiple MIs were older and presented more comorbidities, more frequently underwent invasive testing within the previous 12 months from enrolment such as coronary angiography, and were more aggressively treated with pharmacological therapies compared to patients at their first MI event. These data suggest that the history of multiple recurrent MI is considered by cardiologists as one of the major predictors of risk for cardiovascular diseases, so much so that it deserves more intensive management compared to patients at the first MI.

The prognostic impact of re-MI may be dramatic in patients surviving after a first coronary event [14–19]. Compared to patients without re-MI, those who suffer re-MI showed significantly higher rates of mortality at short- and long-term follow-up [1–9, 14–19]. A recent paper, analyzing a prospective cohort of 3387 patients, showed that re-MI was associated with 25-fold higher risk of death at 1 year compared to patients with a single acute coronary event [20]. This increase in mortality risk may be related, as also suggested by our data, to the greater number of comorbidities present in patients with re-MI rather than to the recurrence of the MI event per se, which appears increasingly rare. Indeed, in recent cohorts of post-MI patients, a progressive decline in re-MI occurrence at long-term follow-up was observed [20, 21]. In our contemporary cohort, which includes only patients with previous MI, it is interesting to note that 13% had a history of 2 MIs and 5% multiple MIs, with an incremental increase in the risk profile.

Observational studies suggested that patients with previous MI and/or myocardial revascularization are less subjected to tests for the evaluation of ischemia and undergo more coronary angiography [22]. This finding was confirmed in our study in which patients with multiple MI more frequently underwent coronary angiography in the year prior to enrolment than patients at their first MI event, even if those with multiple MIs did not present a more extensive coronary artery disease compared to those at their first MI episode. In addition, in patients with a history of multiple MIs, a more aggressive pharmacological therapy, including DAPT, beta-blockers, diuretics, and antianginal drugs, was prescribed by cardiologist compared to those with 1 or 2 MIs. Again, this more invasive and aggressive attitude may be related to the greater risk perceived by cardiologists based on the number of comorbidities rather than the number of previous MI episodes. In this regard, prior studies found incomplete prescription of recommended medications to predict re-MI [23, 24]. In our series, about one in four patients, regardless of the number of prior MIs, was not prescribed OMT at the time of enrolment. This indicates that there was potential to intensify treatment and possibly some of the recurrent MI events could have been delayed or prevented.

### 4.1. Study Limitations

Our study must be evaluated in the light of the known limitations of observational, cross-sectional studies. In addition, even if the participating centers were asked to include in the registry all consecutive post-MI patients, we were not able to verify the enrolment process, due to the absence of administrative auditing. We believe that it is unlikely, however, that selective enrolment in few sites may have substantially changed the study results. Finally, data reported in the present analysis are limited to the time of enrolment, and we do not have data on long-term persistence to prescribed therapies, their changes, and relative outcomes.

## 5. Conclusions

This contemporary, nationwide, real-world cohort of consecutive patients with prior MI managed by cardiologists suggests that those with multiple MIs present an incremental clinical risk, more frequently undergo coronary angiography, and are more intensively managed with pharmacological therapies compared to patients at their first MI episode. Whether this more aggressive attitude is associated with an improved long-term prognosis should be evaluated in large, randomized studies.

## Figures and Tables

**Table 1:** Baseline clinical characteristics, hemodynamic, and laboratory variables of patients presenting with a history of 1, 2, or ≥3 MIs.

	Total population n = 1618	History of 1 MI n = 1335	History of 2 MIs n = 209	History of ≥3 MIs n = 74	p value
Age (years), mean ± SD	66 ± 12	65 ± 12	69 ± 11	70 ± 11	<0.0001
Females, n (%)	316 (19.5)	269 (20.2)	37 (17.7)	10 (13.5)	0.29
Type of last MI STEMI NSTEMI	821 (50.7) 797 (49.3)	718 (53.8) 617 (46.2)	74 (35.4) 135 (64.6)	29 (39.2) 45 (60.8)	<0.0001
Time from last MI 12–24 months 24–36 months	1018 (62.9) 600 (37.1)	843 (63.2) 492 (36.8)	126 (60.3) 83 (39.7)	49 (66.2) 25 (33.8)	0.61
BMI (kg/m^2^), mean ± SD	27.2 ± 4.1	27.2 ± 4.0	27.5 ± 4.3	27.7 ± 4.6	0.29
Active smokers, n (%)	307 (19.0)	254 (19.0)	36 (17.2)	17 (23.0)	0.55
Diabetes mellitus, n (%)	453 (28.0)	346 (25.9)	80 (38.3)	27 (36.5)	0.0003
Hypertensiona, n (%)	1270 (78.5)	1025 (76.8)	176 (84.2)	69 (93.2)	0.0004
Hypercholesterolemia, n (%)	1203 (74.4)	977 (73.2)	166 (79.4)	60 (81.1)	0.06
History of atrial fibrillation, n (%)	207 (12.8)	149 (11.2)	37 (17.7)	21 (28.4)	<0.0001
Chronic renal dysfunction, n (%)	198 (12.2)	132 (9.9)	48 (23.0)	18 (24.3)	<0.0001
Peripheral artery diseasec, n (%)	111 (6.9)	76 (5.7)	26 (12.4)	9 (12.2)	0.0003
COPD¸ n (%)	184 (11.4)	132 (9.9)	38 (18.2)	14 (18.9)	0.0002
Previous stroke/TIA, n (%)	68 (4.2)	43 (3.2)	17 (8.1)	8 (10.8)	<0.0001
History of major bleedings, n (%)	48 (3.0)	34 (2.6)	10 (4.8)	4 (5.4)	0.09
History of heart failure, n (%)	249 (15.4)	160 (12.0)	57 (27.3)	32 (43.2)	<0.0001
Prior PCI, n (%) >2 stent implanted, n (%) available for 1411 pts	1443 (89.2) 353 (25.3)	1185 (88.8) 229 (20.0)	189 (90.4) 72 (39.8)	69 (93.2) 52 (81.3)	0.40 <0.0001
Prior CABG, n (%)	170 (10.5)	98 (7.3)	45 (21.5)	27 (36.5)	<0.0001
Ejection fraction (%), mean ± SD available for 1461 (90.3%) pts	52.4 ± 9.9	53.4 ± 9.0	48.3 ± 12.1	47.3 ± 12.1	<0.0001
SBP (mmHg), mean ± SD	130 ± 17	130 ± 17	127 ± 17	133 ± 21	0.02
Target values of blood pressuree, n (%)	620 (38.3)	497 (37.3)	97 (46.4)	26 (35.1)	0.03
Pulse pressure (mmHg), mean ± SD	53.1 ± 14.3	53.0 ± 14.0	52.3 ± 14.4	56.8 ± 17.4	0.06
HR (bpm), mean ± SD	68 ± 12	67 ± 11	67 ± 13	69 ± 15	0.32
Hb (gr/dl), mean ± SD available for 1099 (67.9%) pts	13.7 ± 1.8	13.8 ± 1.7	13.1 ± 1.9	13.0 ± 2.1	<0.0001
Creatinine (mg/dl), mean ± SD available for 1106 (68.4%) pts	1.1 ± 0.4	1.0 ± 0.4	1.2 ± 0.5	1.3 ± 1.2	0.0001
Total cholesterol (mg/dl), mean ± SD available for 998 (61.1%) pts	145.3 ± 35.6	144.8 ± 35.1	148.5 ± 40.0	146.8 ± 33.6	0.54
LDL cholesterol available for 893(55.2%) pts	76.4 ± 29.4	75.8 ± 28.7	79.2 ± 33.8	80.3 ± 28.7	0.61
Triglycerides (mg/dl), median (IQR) available for 968 (59.8%) pts	106 [80–145]	104 [80–144]	115 [92–156]	105 [75–130]	0.12
Glycemia (mg/dl), mean ± SD available for 980 (60.6%) pts	111.9 ± 32.4	110.2 ± 29.7	120.2 ± 38.8	119.5 ± 49.5	0.01

BMI: body mass index; COPD: chronic obstructive pulmonary disease; Hb: hemoglobin; HR: heart rate; LDL: low density lipoprotein; SBP: systolic blood pressure; STEMI: ST-elevation myocardial infarction; TIA: transient ischemic attack. ^a^ Systolic blood pressure ≥140 mmHg or diastolic blood pressure ≥90 mmHg or use of blood pressure lowering drugs. ^b^ Dialysis, history of renal transplant or creatinine levels >1.5 mg/dL. ^c^History of claudication; amputation for arterial insufficiency; aorta-iliac occlusive disease reconstruction surgery; peripheral vascular bypass surgery, angioplasty, or stent; documented abdominal aortic aneurysm, aneurysm repair or stent; and documented positive noninvasive testing such as abnormal ankle-brachial index or pulse volume recording.^d^ Clinically evident bleeding with haemoglobin reduction ≥ 2 g/dL or requiring transfusion or hospitalization. ^e^ SBP ≤ 120 mmHg and DBP ≤ 80 mmHg

## Data Availability

The data used to support the findings of this study are available from the corresponding author upon request.

## Appendix

Drugs not reported have been used in less than 8% of the cases

Steering committee and list of participating centers and investigators.

Steering Committee.

L De Luca (Chairman), MM Gulizia (Co-Chairman), F Colivicchi, A Di Lenarda, *D* Gabrielli, G Geraci, F Nardi, R Rossini.

Coordinating Center.

ANMCO Research Center (AP Maggioni, *D* Lucci, A Lorimer, G Orsini, L Gonzini, P Priami).

Participating Centers and Investigators.

Salerno (F Piscione, A Silverio, RM Benvenga); Caserta, A.O. S. Anna e S. Sebastiano (F Mascia, A Fusco, S Cicala); Pavia, Fondazione IRCCS Pol. S. Matteo, (L Oltrona Visconti, B Marinoni, U Canosi); Napoli, AOU Federico II (P Cirillo, B Trimarco, F Ziviello); Rimini (D Grosseto, M Menozzi, *D* Mezzena); Napoli, AORN Cardarelli, Cardiologia c/UTIC (C Mauro, A Sasso, A Bellis); Napoli, AORN Osp. dei Colli-PO V. Monaldi (P Calabrò, F Gragnano, A Cesaro); Roma, S. Pertini (V Venturelli, V Porretta, N Borrelli); Catanzaro, Materdomini (C Indolfi, S De Rosa, *D* Torella); Milano, Niguarda, Cardiologia 1 (N Morici); Genova, Galliera (P Bernabò, M Molfese, F Della Rovere); Monfalcone (G Lardieri, T Caiffa, G Moretto); Prato (G Grippo, E Di Vincenzo); Tivoli (A Granatelli, L Lucisano, M Pennacchi); Gorizia (G Lardieri, T Caiffa); Palermo, Villa Sofia-Cervello (G Geraci, N Sanfilippo, A Ledda); Trieste (A Di Lenarda, A Cherubini, G Russo); Frattamaggiore (F Piemonte, A Di Donato, A Carraturo); Benevento, Sacro Cuore di Gesù FBF (B Villari, Q Ciampi, C Contaldi); Rozzano (V Pacher, E Corrada, *D* Cattani); Saronno (D Nassiacos, S Meloni, B Barco); Trento (R Bonmassari, A Bertoldi, F Tedoldi); Andria (M Cannone, G Valenti, RL Musci); Bari, San Paolo (P Caldarola, N Locuratolo, L Sublimi Saponetti); Brescia (L Gentili, C Maiandi); Chieti (M Caputo, CA Capparuccia); Milano, Maggiore Policlinico (T Tonella, FM Massari); Verbania (A Lupi, M Tessitori, M Montano); Milano, S.M. Nascente (A Scaglione, A Torri); Reggio Emilia (G Tortorella, A Navazio); Bolzano (R Cemin, L Latina); Castellanza (D Briguglia, R Marino); Lumezzane (S Scalvini, E Zanelli); Montescano (V Paganini, G Riboni); Pordenone (E Leiballi, A Della Mattia); Rovereto (F Imperadore); Seriate (M Tespili, G Santangelo); Borgomanero (U Parravicini, P Dellavesa); Cecina (R Testa, E Venturini); Fossano (M Feola, M Testa); Lentini (V Crisci, M Tramontana); Lido di Camaiore (L Robiglio); Rivoli (F Varbella, The authors Meynet); Roma, Villa Betania (A Galati, A Maddaluna); Arzignano (C Bilato, The authors Loddo); Augusta (G Licciardello, L Cassaniti); Benevento, G. Rummo (M Scherillo, *D* Formigli); Castel Volturno (L Marullo, L Chianese); Corato (C Paolillo, APA De Santis); Foggia (ND Brunetti, *D* Bottigliero); Roma, Casilino (R Della Bona, MB Giannico); San Donato Milanese, IRCCS, Cardiologia Riabilitativa (R Tramarin, S Lucibello); Ancona, Riuniti (GP Perna, M Marini); Campobasso (AR Colavita); Catania, Garibaldi-Nesima (MM Gulizia, GM Francese); Cuggiono (M Mariani, F Collauto); Magenta (M D'Urbano, R Naio); Messina (G Andò, F Saporito); Milano, Monzino (EM Assanelli, A Cabiati); Paola (A Crivaro, S Alberti); Rieti (The authors Marchese); Roma, Clinica Città di Roma (T Nejat, S Refice); Roma, PO S. Filippo Neri, Cardiologia e UTIC (F Colivicchi, A Aiello); Roma, PO S. Filippo Neri, Card. Riab.-PO Salus (A Galati, GR Cristinziani); Roma, Umberto Primo (F Barillà, R Iorio); Sanremo (G Mascelli, SN Tartaglione); Santa Maria Capua Vetere (G Di Chiara, *D* D'Andrea); Ancona, INRCA (R Antonicelli, G Malatesta); Firenze (C Di Mario, A Mattesini); Spoleto (L Tramontana, S Conti); Viterbo (L Sommariva, A Celestini); Catania, Cannizzaro (F Amico, S Giubilato); Galatina (AF Amico, M De Filippis); Gavardo (GF Pasini, M Triggiani); Manfredonia (V Ferrara, S Cappetti); Milano, San Paolo (S Carugo, S Lucreziotti); San Benedetto Del Tronto (M Persico, G Gizzi); Cefalù (T Cipolla, A Caronia); Fidenza (E Buia, P Pastori); Foligno (M Scarpignato, E Biscottini); Legnano (F Poletti, C Vimercati); Milano, Niguarda, Cardiologia 4 (R Pirola); Negrar (E Barbieri, C Dugo); Osio Sotto (N De Cesare, ML De Benedictis); Reggio Calabria, Madonna della Consolazione (A Ruggeri); San Fermo della Battaglia (C Campana, S Bonura); San Giovanni Rotondo (C Vigna, N Marchese); Sondalo (NG Partesana, P Bandini); Cassano delle Murge (G Farinola, *D* Santoro); Catanzaro, Pugliese (F Cassadonte); Empoli (F Calabrò, M Sansoni); Erice (MG Abrignani, F Bonura); Fermo (D Gabrielli, M Benvenuto); Lecce (A Liso, T Passero); Milano, CTO (The authors Mori, B Pozzoni); Roma, S.G. Addolorata (F Prati, ML Finocchiaro); Sorrento (N Tufano); Melito di Porto Salvo (B Miserrafiti, V Lacquaniti); Mestre, San Marco (F Del Piccolo, B Mohamad); Moncalieri (MT Spinnler, V Bovolo); Palermo, Casa di Cura Candela (E Rebulla, M Pieri); Pescara (L Paloscia, *D* Di Clemente); Piossasco (G Mazzucco, A Micanti); Ponte San Pietro (P Peci, O Ornago); Roma, Aurelia Hospital (F Proietti, M Michisanti); Scandiano (A Reverzani, A Donatini); Avola (P Costa, S Russo); Belluno (E Franceschini Grisolia, L Mario); Boscotrecase (F Di Palma, F Dell'Aquila); Busto Arsizio (A Maestroni, SI Caico); Castellammare di Stabia (G De Caro, L Attianese); Esine (S Perotti, V Cotti Cometti); Genova, Padre Antero Micone (D Astengo); Guastalla (A Navazio, E Guerri); Milano, S. Raffaele (D Cianflone, F Maranta); Napoli, Fond. Evangelica Betania (N Esposito, M Malvezzi Caracciolo D'Aquino); Nola (L Caliendo, C Ricci); Reggio Calabria, Bianchi Melacrino Morelli (CP Ceruso, S Lanteri); Roma, S. Pietro FBF (R Serdoz, E Bruno); San Felice a Cancello (C De Matteis, C Campagnuolo); San Giuseppe Vesuviano (MA Ammirati, VM Corrado); Arco (MA Amado Eleas); Aversa (L Fattore); Avezzano (C Ippoliti); Conegliano (G Turiano); Feltre (C Piergentili); Gallarate (SI Caico); Genova, S. Martino (F Chiarella); Napoli, S.G. Bosco (P Capogrosso); Pavia, ICS Maugeri SPA Società Benefit (M Perotti); Pescia (S Di Marco); Pozzuoli (G Sibilio); Sessa Aurunca (L Di Lorenzo); Taranto (A Aurelio); Vicenza (AB Ramondo); Bari, Policlinico (D Zanna); Castelfranco Veneto (C Cernetti); Giugliano in Campania (G Napolitano); Imola (S Negroni); Latina (N Alessandri); Mestre, Dell'Angelo (F Rigo); Molfetta (F Giusti); Nuoro (G Casu); Peschiera Del Garda (A Vicentini); Policoro (G Calculli); Pomezia (MS Fera); Vittoria (GV Lettica); Volterra (G Vagheggini); Bergamo (A Pitì); Caserta, Villa del Sole (A Porfidia); Ciriè (A Di Leo); Ivrea (A Ravera); Licata (E Ciotta); Mirano (S Saccà); Napoli, AORN Cardarelli, Cardiologia Generale c/Riabilitazione (O Silvestri); Piombino (S Isidori); S. Omero (P Natali); San Bonifacio (M Anselmi); San Donato Milanese, IRCCS, Cardiologia c/UTIC (L Testa); Sesto San Giovanni (A Antonelli); Sondrio (E Tavasci); Telese (G Furgi); Teramo (A Lavorgna); Treviso (N Gasparetto); Udine (T Bisceglia).

## Abbreviations

ACE: Angiotensin-converting enzyme inhibitors
ARB: Angiotensin receptor blockers
CCB: Calcium channel blockers
DAPT: Dual antiplatelet therapies
MRA: Mineralocorticoid receptor antagonists
OAC: Oral anticoagulants
OMT: Optimal medical therapy.

## References

[1] Dalen J. E., Alpert J. S., Goldberg R. J., Weinstein R. S. (2014). The epidemic of the 20th century: coronary heart disease. *The American Journal of Medicine*.

[2] Yeh R. W., Sidney S., Chandra M., Sorel M., Selby J. V., Go A. S. (2010). Population trends in the incidence and outcomes of acute myocardial infarction. *New England Journal of Medicine*.

[3] Marques-Vidal P., Ruidavets J. B., Cambou J. P., Ferrières J. (2000). Incidence, recurrence, and case fatality rates for myocardial infarction in southwestern France, 1985 to 1993. *Heart*.

[4] Messner T., Lundberg V., Boström S., Huhtasaari F., Wikström B. (2003). Trends in event rates of first and recurrent, fatal and non-fatal acute myocardial infarction, and 28-day case fatality in the Northern Sweden MONICA area 1985 - 98. *Scandinavian Journal of Public Health*.

[5] McGovern P. G., Jacobs D. R., Shahar E. (2001). Trends in acute coronary heart disease mortality, morbidity, and medical Care from 1985 through 1997. *Circulation*.

[6] Buch P., Rasmussen S., Gislason G. H. (2007). Temporal decline in the prognostic impact of a recurrent acute myocardial infarction 1985 to 2002. *Heart*.

[7] Brown T. M., Deng L., Becker D. J. (2015). Trends in mortality and recurrent coronary heart disease events after an acute myocardial infarction among Medicare beneficiaries, 2001-2009. *American Heart Journal*.

[8] Chaudhry S. I., Khan R. F., Chen J. (2014). National trends in recurrent AMI hospitalizations 1 year after acute myocardial infarction in medicare beneficiaries: 1999-2010. *Journal of American Heart Association*.

[9] Luca L. D., Paolucci L., Nusca A. (2021). Current management and prognosis of patients with recurrent myocardial infarction. *Reviews in Cardiovascular Medicine*.

[10] De Luca L., Piscione F., Colivicchi F. (2018). Contemporary management of patients referring to cardiologists one to three years from a myocardial infarction: the EYESHOT Post-MI study. *International Journal of Cardiology*.

[11] Ibanez B., James S., Agewall S. (2018). ESC guidelines for the management of acute myocardial infarction in patients presenting with ST-segment elevation. *European Heart Journal*.

[12] Roffi M., Patrono C., Collet J.-P. (2016). 2015 ESC Guidelines for the management of acute coronary syndromes in patients presenting without persistent ST-segment elevation. *European Heart Journal*.

[13] De Luca L., Colivicchi F., Meessen J. (2019). EYESHOT Post-MI Investigators. How do cardiologists select patients for dual antiplatelet therapy continuation beyond 1 year after a myocardial infarction? Insights from the EYESHOT Post-MI Study. *Clinical Cardiology*.

[14] Krumholz H. M., Normand S.-L. T., Wang Y. (2019). Twenty-year trends in outcomes for older adults with acute myocardial infarction in the United States. *JAMA Network Open*.

[15] Hanssen M., Cottin Y., Khalife K. (2012). French registry on acute ST-elevation and non ST-elevation myocardial infarction 2010. FAST-MI 2010. *Heart*.

[16] García-García C., Oliveras T., Serra J. (2020). Trends in short- and long-term ST-segment-elevation myocardial infarction prognosis over 3 decades: a mediterranean population-based ST-segment-elevation myocardial infarction registry. *Journal of American Heart Association*.

[17] Culler S. D., Kugelmass A. D., Cohen D. J. (2019). Understanding readmissions in medicare beneficiaries during the 90-day follow-up period of an acute myocardial infarction admission. *Journal of American Heart Association*.

[18] Sreenivasan J., Abu-Haniyeh A., Hooda U. (2020). Rate, causes, and predictors of 90-day readmissions and the association with index hospitalization coronary revascularization following non-ST elevation myocardial infarction in the United States. *Catheterization and Cardiovascular Interventions*.

[19] Smolina K., Wright F. L., Rayner M., Goldacre M. J. (2012). Long-term survival and recurrence after acute myocardial infarction in England, 2004 to 2010. *Circulation: Cardiovascular Quality and Outcomes*.

[20] Song J., Murugiah K., Hu S. (2021). Incidence, predictors, and prognostic impact of recurrent acute myocardial infarction in China. *Heart*.

[21] Wang Y., Leifheit E., Normand S. T., Krumholz H. M. (2020). Association between subsequent hospitalizations and recurrent acute myocardial infarction within 1 Year after acute myocardial infarction. *Journal of American Heart Association*.

[22] Lin G. A., Dudley R. A., Lucas F. L. (2008). Frequency of stress testing to document ischemia prior to elective percutaneous coronary intervention. *JAMA*.

[23] Huber C. A., Meyer M. R., Steffel J., Blozik E., Reich O., Rosemann T. (2019). Post-myocardial infarction (MI) Care: medication adherence for secondary prevention after MI in a large real-world population. *Clinical Therapeutics*.

[24] Silverio A., Benvenga R. M., Piscione F. (2021). Prevalence and predictors of out-of-target LDL cholesterol 1 to 3 Years after myocardial infarction. A subanalysis from the EYESHOT post-MI registry. *Journal of Cardiovascular Pharmacology and Therapeutics*.

